# Continued Discontinuation of TKI Treatment in Medullary Thyroid Carcinoma – Lessons From Individual Cases With Long-Term Follow-Up

**DOI:** 10.3389/fendo.2021.718418

**Published:** 2021-09-29

**Authors:** Tim Brandenburg, Vera Tiedje, Philipp Muchalla, Sarah Theurer, Frank Weber, Kurt Werner Schmid, Henning Dralle, Dagmar Führer

**Affiliations:** ^1^ Department of Endocrinology, Diabetology and Metabolism, Endocrine Tumour Center at West German Cancer Center, University of Duisburg-Essen, Essen, Germany; ^2^ Department of Medicine and Human Oncology and Pathogenesis Program, Memorial Sloan Kettering Cancer Center, New York, NY, United States; ^3^ Institute of Pathology, University Hospital Essen, University Duisburg-Essen, Essen, Germany; ^4^ Department of General, Visceral and Transplantation Surgery, Section of Endocrine Surgery, University Hospital Essen, University of Duisburg-Essen, Essen, Germany

**Keywords:** medullary thyroid carcinoma, multiple endocrine neoplasia type 2a, tyrosine kinase inhibitor therapy, therapy discontinuation, calcitonin doubling time

## Abstract

**Background:**

The tyrosine kinase inhibitors (TKI) vandetanib and cabozantinib are approved as targeted therapies in advanced medullary thyroid carcinoma (MTC) with symptoms or high tumour burden. Only recently, toxicity in long-time TKI usage was analysed. However, little is known about the impact of TKI discontinuation on MTC disease course after longer-term therapy. Here, we report our experience in a series of 7 MTC patients with vandetanib treatment of up to 87 months followed by discontinuation for concerns of toxicity or due to side-effects. The discontinuation of TKI therapy is a relevant clinical scenario. To our knowledge we present the largest single center series on an important aspect of TKI management.

**Methods:**

Retrospective analysis of MTC patients with continued discontinuation of vandetanib treatment in a tertiary referral endocrine tumour centre. Analysis included a review of patients’ records for TKI indication, and treatment response as well indications for continued TKI discontinuation and follow-up by clinical assessment, calcitonin and CEA doubling times as well as imaging (ultrasound, CT).

**Results:**

Seven MTC patients [6 sporadic MTC, 1 Multiple Endocrine Neplasie Type 2a (MEN2a)] with previous vandetanib treatment (median: 41 months; range 7-87 months) and continued TKI discontinuation were identified out of 161 analysed MTC files. TKI treatment was initiated due to high tumour burden and symptoms or RECIST (Response Evaluation Criteria In Solid Tumors) progression in all patients. Two patients (29%) remained stable after discontinuation of vandetanib until now (follow-up of 47 and 61 months). Both patients had been on TKI therapy for 73 and 58 months. Five patients (71%) developed progressive disease after TKI discontinuation. In 2 patients, vandetanib was restarted after 45 and 52 months resulting again in disease control. One patient was enrolled in a new RET kinase inhibitor trial after 45 months of vandetanib discontinuation. Two patients declined restart of treatment due to mental health issues leading to discontinuation of vandetanib in the first place (after 7 and 38 months of treatment) and both patients died of rapidly progressive disease. At time points of tumour progression, calcitonin-doubling time (CDT) was < 2 years in all patients.

**Conclusion:**

This case series suggests that discontinuation of long-term vandetanib treatment with documented stable disease does not automatically result in rapid disease progression but may be followed by prolonged “TKI free” stable disease in individual patients. Analysis of calcitonin and CDT during discontinuation is indicated as it will unmask tumour progression earlier than imaging. Restart with the same TKI is possible in case of progression.

## Introduction

Medullary thyroid cancer (MTC) arises from parafollicular cells of the thyroid gland and is a rare neuroendocrine tumour (1–3). MTC occurs sporadically in about 75% and is inherited as part of the Multiple Endocrine Neoplasia Type 2 (MEN2) syndrome in 25% of cases (4). Both conditions are associated with genetic alterations in the *rearranged during transfection (RET)* proto-oncogen, with somatic *RET* mutations found in sporadic MTC and germline *RET* mutations leading to MEN2 (5–10).

Calcitonin functions as a diagnostical tool and marker of tumour burden. In addition calcitonin (CDT) as well as CEA doubling times (CEA-DT) have been established as important prognostic markers for MTC disease course and together with imaging are used to assess the biological course in case of metastasised MTC (2, 11, 12).

In Germany, two drugs, vandetanib and cabozantinib, have been approved for targeted therapy in advanced MTC in 2012 and 2014, respectively based on randomised phase III trials (13, 14). Since advanced MTC can remain stable and free of symptoms over a long period of time, treatment indication should be thoroughly evaluated *versus* the concept of “wait and see”. Treatment indication has been licensed for *aggressive* (high tumour burden and/or high tumour dynamics with RECIST progression (>20%) within 6-12 months*) and symptomatic medullary thyroid cancer (MTC) in patients with unresectable locally advanced or metastatic disease* and it is recommend to continue treatment as long as response and patient benefit is present (15–17).

Only recently, Ramos et al. (18) reported an ensuring safety profile of long-term TKI therapy including 76 vandetanib treated MTC patients (overall median treatment duration was 17.6 (range: 0.7-130.6) months; 21/76 (27.6%) patients received vandetanib for more than 48 months (classified as long-term user) with a median treatment duration of 68.1 (range: 49.1-130.6) months) (18). However, some patients question continued TKI treatment over many years or may require treatment discontinuation due to severe side effects. In these situations, little is known about the natural course of disease. Here we report the outcome in a retrospective analysis of MTC patients at our centre with vandetanib discontinuation because of long-term usage, severe side-effects or patient’s refusal to continue treatment.

## Material and Methods

Patients included in this retrospective analysis were six sporadic and one hereditary MTC patients of our department with documented TKI treatment discontinuation after vandetanib therapy either as part of a clinical trial or after vandetanib approval (**Tables 1**, **3**). In total, 161 MTC patient records were analysed. Pathologic confirmation of MTC had been obtained in all cases. *RET* mutation analysis was performed retrospectively in the sporadic cases by using next-generation sequencing but were not provided in all cases due to consistency of the tumour sample. Patients’ files were reviewed for primary TKI treatment indication and TKI response. Pretreatment and follow-up staging under vandetanib included computed tomography (CT) of neck, chest, brain and abdomen, initially at 3 months, later at 6-12months intervals. During TKI discontinuation patients were followed up clinically, by imaging (ultrasound, CT) and determination of calcitonin levels. Calcitonin levels before TKI start, before TKI discontinuation and at the indicated endpoints (progressive disease (PD) or end of observation (04/2021) were documented.

Vandetanib was given orally at doses of 300 mg once daily and 200 mg daily. Patient characteristics and treatment courses are shown in **Tables 1**, **3**. A summary of gender, age at diagnosis, mutation analysis and initial tumour burden is shown in **Table 2**.

**Table 1:** Course of disease in 6 patients with sporadic medullary thyroid carcinoma (sMTC) and in 1 patient with MEN2a.

Patient	TKI	Status at TKI-initiation	Tumour marker prior to TKI-initiation Calcitonin [pg/ml] /CDT (years) CEA [ng/ml]/ CEA-DT [years]	Calcitonin-Level before TKI-pausation	Reason for TKI-pausation/CTCAE	Calcitonin /CEA after ≥3 months of pausation	TKI treatment [months]	Time of TKI pausation to progression or end of observation in 04/2021 [months]	Calcitonin/CDT and CEA at progression or end of observation (04/2021)
1	Vandetanib	PD (LN)	4850/ 0.83 CEA: n/a	1532/ 11,34 CEA: 29,2/ n/a	Therapy duration	3344 CEA: 44,6	73	47	SD, 9650/3,78 max. 13575 CEA: 66/3,83
2	Vandetanib	High tumour burden with dynamics in the past, M (hep)	19361/ 4,72 CEA: 1050/ 3,42	6523/ 7,35	Therapy duration	3951 CEA: 134	58	61	SD, 9397/ 7,45 CEA: 326
3	Vandetanib	M (pul),PD M (pul)	526/ 2,73 CEA: 19.1/ n/a	175/-0,52 CEA: 5,3/-0,33	Psychological stress, patient´s request	270 CEA: 5,1	7	9	PD, 877/0,3 CEA: 21,9/0,25 Death 6 months after PD; 15 months (01/15) after TKI discontinuation
4	Vandetanib	PD (LN)	1923/1,94 CEA: 10,2 /n/a	276/ 22,4 CEA: 12,2/ n/a	Therapy duration	946 CEA: 18,3	87	45	PD (pul, oss, LN), 4128/ 1,57 CEA: 39,6/2,54 restart TKI, now SD
5	Vandetanib	PD (LN), M (pul, cer)	26,040/ 9,64 CEA: 372,3/ 17,84	14806/2,45 CEA: 121.1/ -1,02	Abscess (thigh) CTCAE III, patient´s request	15,820 CEA: 522	12	52	PD, 15751/ 17,26 CEA: 729/ 2,1
6	Vandetanib	PD M (pul, oss, cer)	216/ 1,76 CEA: 2,7/ 0,97	38,5/ 0,66 CEA <2	ECOG	188 CEA<2	38	5	PD, 188/ 0,29 CEA: <2 Death 2 months after PD; 7 months after TKI discontinuation
7.	vandetanib	PD M (pul, oss)	5087/ n/a CEA: 154/ n/a	61,7 CEA: 11,4	Meningitis & myocarditis	110 CEA: 9,9	36	45	PD 9222/ 1,1 CEA: 87/0,8

Indication for TKI therapy with vandetanib. Calcitonin level at the beginning and before TKI discontinuation as well as 3 months after TKI pausation SD, stable disease; PD, progressive disease; n/a, data not available; pul, pulmonal; oss, bone; LN, lymphonodual; cer, cerebral, CDT, calcitonin doubling time; CEA, carcinoembryonal antigen; CEA DT, CEA doubling time; CTCAE, common terminology criteria for adverse events; ECOG, Eastern Cooperative Oncology Group performance status.

**Table 2:** Patient characteristics.

Patient	Gender	Age at diagnosis	TNM	pathology	RET analysis
1	Female	41	pT3, pN2(14/27), cM0, G2, pL0, pV0, R0	sMTC	M918T (sporadic)
2	Male	38	pT2a pN1 (3/10) cM1(oss)	sMTC	no material available
3	Female	45	pT3 pN1b cM0	sMTC	no material available
4	Female	19	pT4 pN1b M0	sMTC	M918T (sporadic)
5	Female	43	pT3b pNx pMx R1	sMTC	no material available
6	Female	56	pT2 pN0 (0/29)	sMTC	no material available
7	Male	23	pT1m pN1b (17/36) pM1 (hep,oss)	MEN2a	L790F Germline

Age of diagnosis, gender, initial TNM-classification and molecular pathology. sMTC, sporadic medullary thyroid cancer; MEN2A, Multiple Endocrine Neoplasia Type 2A.

**Table 3:** Summarised course of disease in 6 patients with sporadic medullary thyroid carcinoma (sMTC) and in 1 patient with MEN2a.

Patient	Period elapsed after MTC diagnosis [months]	Tumor burden/Progress according to RECIST	ECOG status at therapy initiation	Therapy duration[months]	Reason to discontinue vandetanib	Therapy-pause duration until progression [months]	CDT at progression or end of observation	Intervention	Status
**1**	252	Moderate / yes	0	73	Duration *vs.* concern about toxicity	Ongoing (47 months, SD)	> 2	–	SD
**2**	76	High / no	0	58	Duration *vs.* concern about toxicity	Ongoing (61 months, SD)	> 2	–	SD
**3**	102	High / yes	0	7	Patient´s will, AE	9 (PD)	< 2	Re-start declined	Dead 6 months after PD; 15 months after TKI pausation
**4**	47	Moderate / yes	0	87	Duration *vs.* concern about toxicity	45 (PD)	< 2	Re-start vandetanib	SD in latest scan 29 months after re-start of TKI
**5**	87	High / yes	0	12	AE	52 (PD)	>2, but CEA-DT critical (2.1 yrs)	Re-start vandetanib,	SD in latest scan 12 months after re-start of TKI
**6**	54	High / yes	1	38	AE	5 (PD)	< 2	Re-start declined	Dead 2 months after PD; 7 months after TKI pausation
**7**	31	High / yes	0	36	AE	45 (PD)	< 2	Enrollment in clinical trial	SD

Indication for TKI therapy with vandetanib. Tumour burden (high: progressive distant metastasis; moderate: progressive locoregional metastasis), therapy duration, reason to discontinue therapy, therapy pause duration until progression or end of observation in 04/2021 along with CDT and current status are shown. SD, stable disease; PD, progressive disease; CDT, calcitonin doubling time; CEA-DT, carcinoembrionic antigen doubling time; AE, Adverse event.

## Results

In all seven patients vandetanib treatment had been initiated for progression of MTC according to RECIST (six patients with inoperable locoregional disease and/or distant metastasis) or due to high tumour burden with documented progression in the past (patient 2).

Two patients presented with progressive disease, and TKI therapy was restarted. Two patients paused therapy due to individual patient’s will or side effects and did not restart therapy. Two patients remained stable after pausation.

Patient 1 was diagnosed with sporadic MTC at the age of 41. Initial therapy comprised thyroidectomy and neck dissection (no detailed data available before 1990). TNM stage was pT3, pN2(14/27), cM0, pL0, pV0, R0. The patient underwent recervicotomy 2 and 5 years later. Eighteen years after MTC diagnosis, DOTATOC-PET/CT showed SSTR-positive cervical and axillary lymph node metastases. One year later, a single liver metastasis was resected in toto. During this time calcitonin level rose from 371 pg/ml to 1,133 pg/ml resulting in a CTD < 2 years. New bone lesions were diagnosed in the following year and zoledronate treatment was commenced. In 2011, 21 years after initial MTC diagnosis, CDT was 10 months, CEA was rising (47,2 ng/ml), and imaging showed progressive disease (bone, lymph nodes). The patient was enrolled in the D4200C00088 trial (“A Randomized, International, Open-Label, Multi-Centre, Phase III Study to Assess the Effect of a Patient Outreach Program on the Percentage of Time Patients with Locally Advanced or Metastatic Medullary Thyroid Cancer Experience Grade 2 or Higher Adverse Events during the First 12 Months of Treatment with Vandetanib.”) (19). The first staging three months after start of vandetanib showed disease stabilisation and the patient remained on TKI therapy for 73 months with stable disease and manageable adverse events (diarrhoea CTCAE 3, Fatigue CTCAE 2). After this time vandetanib was discontinued as an informed patient’s decision due to long-term disease stabilisation. The patient was closely monitored at 3 months follow-up visits. No structural progression was detected by imaging until now over 47 months, even though calcitonin and CEA rose from 1,532 pg/ml to max 13,575 pg/ml (CDT 3,78 years) and 29,2 ng/ml to max 66,9 (CEA-DT 3,83 years), respectively. The patient is asymptomatic. Molecular analysis revealed a somatic M918T mutation in the tumour tissue.

Patient 2 was diagnosed with sporadic MTC at the age of 38. His initial therapy consisted of thyroidectomy and lymphadenectomy (TNM stage pT2, pN1 (3/10), cM1). Postoperatively, Calcitonin-levels persisted at 7,486 pg/ml and CEA at 292 ng/ml. Aside from multiple bone metastases (vertebrae and pelvis), liver metastases were detected in subsequent scans, accompanied by rise in CT and CEA levels. In 2010, 5,5 years after diagnosis, the patient suffered a pathological bone fracture. Seven years after diagnosis and with substantial tumour burden the patient was enrolled in the D4200C00088 trial (calcitonin level of 19,361 pg/ml and CEA of 1,050 ng/ml at therapy start). Under vandetanib disease stabilisation was documented on subsequent scans and seven months after TKI start, local treatment of bone metastases by chemoembolisation (cervical vertebrae 7) was performed. After 58 months on vandetanib with documented stable disease, the decision was made to pause TKI therapy, analogously to patient 1 due to – at the time – lacking information on long-term toxicity in a young patient. Follow-Up since then showed stable disease at 61 months after vandetanib discontinuation with constant calcitonin-levels (last follow-up 04/2021: 9,397 pg/ml and CEA 326 ng/ml. CDT 7,45 years). No *RET-*protooncogene mutation was detected in exons 11,10,13-16 in the tumour tissue.

Patient 3 was diagnosed of sporadic MTC at 45-years of age. She underwent total thyroidectomy and neck dissection. TNM stage was pT3, pN1b, cM0. Calcitonin level remained elevated (64 pg/ml) postoperatively. Within the next seven years, CDT remained >2 yrs. and imaging did not show locoregioal or distant disease. One and half year later, CT scanning showed pulmonary and mediastinal lesions, which were histologically confirmed MTC metastases. CDT was < 2 yrs. and due to tumour dynamics, vandetanib therapy was initiated. Three months later, partial response was documented. QTc prolongation CTCAE grade 2 (480ms) was found under vandetanib treatment. After seven months of TKI therapy, the patient required discontinuation because of mental health issues. Imaging after 3 and 6 months showed stable disease with an increasing calcitonin of 270 pg/ml and 554pg/ml. Though restart of vandetanib was strongly recommened at all visits, the patient declined treatment. Nine months after TKI discontinuation, imaging showed brain metastases. At this time point, the patient again refused therapy and wished to discontinue follow-up. She died six months later.

Patient 4 was 19 years old when sporadic MTC (pT4, pN1b, M0) was diagnosed. Five months after thyroidectomy, atypical lung resection was performed, along with radical mediastinal lymphadenectomy. Three years after first diagnosis neck dissection was performed leading to permanent hypoparathyroidism. Seventy-four months after MTC diagnosis the patient was enrolled in the ZETA (13) study (clin.gov.: NCT00410761) due to locoregional progressive disease along with increasing calcitonin (1,923 pg/ml, CDT < 2 years) and CEA (10,2 ng/ml) levels. Stable disease was documented on imaging under vandetanib therapy. After 87 months of TKI treatment, an informed decision was made to pause therapy due to lack of knowledge on long-time cytotoxicity. Four months after vandetanib discontinuation calcitonin increased from 258 pg/ml to 1,643 pg/ml) and CEA rose from 12,7 ng/ml to 20,9 ng/ml. One year later, a new suspicious lesion was detected on ultrasound in the right thyroid bed, with calcitonin at 1,122 pg/ml and CEA at 18,8 ng/ml. No changes were documented at 15- and 21-months follow-up. Twenty-nine months on TKI discontinuation, cholecystectomy due to symptomatic cholecystolithiasis was performed and biopsy of a solitary liver lesion, documented for years as “haemangioma” was performed, which in fact turned out to be a MTC metastasis. Further follow-up imaging did not show progressive disease until 45 months when progressive disease with mediastinal lymph nodes, hepatic and bone metastases was diagnosed. Calcitonin levels had risen to 4,128 pg/ml (CDT < 2 years) and CEA to 39,6. Vandetanib treatment was restarted along with bisphosphonate therapy resulting in partial remission after 4 months. Regular follow-until now (29 months) showed stable disease without severe side effects. Retrospective analysis revealed a somatic *RET* M918T mutation in the tumour tissue.

Patient 5 was diagnosed with sporadic MTC at the age of 43 with initial calcitonin of 12,700 pg/ml and CEA of 286 ng/ml. The patient underwent thyroidectomy, TNM stage was pT3, pNx, cM1, R1. Postoperatively, calcitonin raised to 43,466 pg/ml and recervicotomy was performed. Calcitonin dropped to 37,313 pg/ml. Imaging showed persistend locoregional and distant pulmonal metastases. Due to significant tumour burden with pulmonary metastases and residual local disease, the patient was included in the ZETA trial (13) (clin.gov.: NCT00410761). Stable disease was documented on imaging over 87 months under study medication until imagery revealed progressive disease with bone metastases and a new cerebella metastasis. Unblinding revealed that the patient was in the placebo arm, and he was then crossed over to the vandetanib treatment arm. Three months later vandetanib dosage was reduced to 200 mg/d due to side effects (acne CTCAE 3, hypacusis CTCAE 2 and stomatitis, CTCAE 2). After 12 months of treatment and disease stabilisation the patient required TKI discontinuation due to persisting side effects like diarrhoea and abscess formation of the thigh and axilla with consecutive lancing and antibiotic treatment (CTCAE 3). In the following, radiotherapy was administered for bone metastases (lumbar (total of 3 Gy) and thoracic (total of 30 Gy) vertebrae) and stable disease was observed over 52 months (CDT > 2 years) until lately imaging revealed morphological progression in the patient along with critical CEA-DT of 2.1 years. An informed consent was obtained to restart TKI therapy, with patient’s preference for vandetanib over cabozantinib. Imaging after three months showed partial response and stable disease after six months until now (12 months, last follow-up: 04/2021).

In patient 6, MTC [pT2, pN0 (0/29)] was incidentally diagnosed after hemithyroidectomy for nodular thyroid disease. Postoperative calcitonin after completion surgery was 5,2 pg/ml and remained stable until 44 months, when calcitonin levels increased (23 pg/ml) and imaging by ultrasound and DOTATOC-PET/CT revealed a new suspicious cervical lesion. Surgery failed to identify tumour tissue. Within the next 20 months, calcitonin rose and CDT dropped to 1,63 years. At this time imaging revealed pulmonary, bone and cerebral metastases. Radiotherapy of the brain metastasis was initiated (total of 40 Gy) along with start of vandetanib therapy. Imaging 3 months after TKI start showed partial remission, but due to side effects TKI dose had to be reduced to 200mg/d. Calcitonin level dropped below 2 pg/ml. Radiotherapy of spinal vertebrae was performed (total of 39,6 Gy) after 7 months. After one year on vandatenib treatment dysesthesias in all extremities occurred. Neurological evaluation and imaging did not show myeloncompression and symptoms resolved spontaneously. Besides phototoxicity CTCAE 2, no further events were documented. After 38 months, the patient requested discontinuation of vandetanib therapy due to mental health issues and impaired well-being (ECOG 2-3). Five months after discontinuation, calcitonin level had increased to 188 pg/ml and progression of pulmonary and bone metastases was documented. The patient declined restart of TKI therapy and died two months later.

Patient 7 is the index patient in a MEN2 family (L790F *RET* germline mutation) and was diagnosed with MTC {pT1m, pN1b (17/36), pM1 [hep (liver), oss (bone)]} at age 23. Postoperative calcitonin was 1,918 pg/ml and increased to 3,164 pg/ml within 6 months. Nineteen months after diagnosis, locoregional progression and new bone metastases were diagnosed and the patient underwent neck dissection and radiotherapy. Due to further progressive disease including new lung metastases. 31 months after MTC diagnosis, the patient was included in the ZETA trial (13) (clin.gov.: NCT00410761) (43). Before vandetanib therapy start, calcitonin was 5,087 pg/ml, and CEA was 154 g/ml. Besides acne (CTCAE 2), TKI was well tolerated. Staging after three months showed partially remission, and imaging at 6 and 12 months revealed stable disease. Vandetanib was continued after the end of ZETA trial, bisphosphonate therapy was added, and treatment continued until 36 months when the patient became severely ill and required ICU treatment for meningitis with seizures and myocarditis. The patient had full recovery and MTC disease remained stable despite discontinuation of vandetanib over 37 months and increased calcitonin (7,663 pg/ml) and CEA (51,8 ng/ml). During this time, the patient started a family. Forty-five months after TKI pause progressive disease was documented with a new cerebellar lesion (calcitonin 9,222 pg/ml (CDT 1,1), CEA 87 ng/ml). The patient received stereotactic radiotherapy and was enrolled in the BMS-CA 627-Study (NCT02832167) (20).

## Discussion

Though discontinuation of TKI treatment is not recommended in MTC patients who benefit from treatment, our retrospective analysis shows that cessation of long-term vandetanib therapy with documented stable disease does not automatically result in rapid disease progression but may in fact be followed by prolonged “TKI free” stable disease in individual patients.

So far, recent follow-up studies have mainly addressed the outcome of continuously long-term vandetanib treated MTC patients. For example, out of 76 patients analysed by Ramos and colleagues (18), 21 had been given vandetanib beyond 48 months (defined “long-term”), and 3/21 in fact discontinued therapy (one voluntary and two due to toxicity) however their outcome was not reported. Interestingly, the authors described severe adverse events, like cholecystitis, acute pancreatitis, nephrolithiasis, and posterior encephalopathy, which had newly occurred after 48 months of therapy. Therefore, discontinuation in long-term TKI patients is an important topic.

Additionally, Valerio (21) and colleagues analysed a collective of 24 out of 79 patients, who received vandetanib longer than 48 months and seven patients had to pause the treatment because of a very severe AE (i.e. heart attack, stroke, orbital edema, hypertension, weight loss, asthenia and creatinine increase) but again the patient outcome was not reported, indicating the need for real-world data on MTC course upon TKI discontinuation.

In our series vandetanib was stopped in patients 1,2 and 4 due to patients’ concerns about toxicity *vs.* documented stable disease under long-term TKI therapy (58-87 months, **Table 3**). Patient 1 and 2 remained stable until now (follow-up of 47 and 61 months, CDT > 2 yrs). Patient 4 developed progressive disease after 45 months (CDT < 2 yrs.), but disease stabilisation was obtained after re-start of vandetanib therapy.

In this context, initial TNM stage and tumour mass might play a crucial role in the respective individual outcomes since type of progression was different in the patients when they entered the trials with indications of tumour burden (patients 1 and 2) or rather locoregional progressive MTC (patient 4). Valerio et al. (21) analysed their collective for predictors of a longer and durable response upon vandetanib therapy. In their study *early treatment with vandetanib, when patients are younger* (age < 45 years)*, with a good ECOG performance status and symptomatic disease, not necessarily progressing for RECIST, seemed to be the best predictors of a longer and durable response* (21). This could additionally explain long-term stability upon vandetanib treatment in patients 1,2 and 4 in our study, which subsequently might affect endured stability upon therapy discontinuation (and therapy reinduction in patient 4).

Patients 3, 5 and 6 stopped vandetanib due to personal reasons after 7, 12 and 38 months of therapy. Progressive disease occurred within less than 9 months in 2 patients (3 and 6) who declined further treatment and succumbed to disease. In the other patient (patient 5) progression was documented only after 52 months and restart of vandetanib resulted in stabilisation again. Vandetanib had to be discontinued in patient 7 due to a life-threatening event and this was followed by a period of 37 months of “drug holiday” with stable disease until eventually progression occurred. In this subgroup, CDT was < 2 in almost all patients (in patient 5 with critical CEA-DT instead) before radiological progression.

Taken together, our case series could stimulate discussion on an important aspect of TKI management. Cleary a limitation is the small sample number and further confirmation in larger series is desirable and will be highly clinically relevant. Importantly, monitoring CDT/CEA-DT during TKI discontinuation is of utmost importance and in the case of disease progression, TKI re-start led in our patients to disease stabilisation. Whether *RET* mutation status plays a role is unknown. This was not feasible in our analysis but might be interesting.

Fortunately, RET kinase inhibitors have been approved meanwhile and provide novel options in patients that require change in targeted therapy for personal reasons or side effects but the issues on effective treatment duration remain open (22, 23).

## Data Availability Statement

The original contributions presented in the study are included in the article/supplementary material. Further inquiries can be directed to the corresponding author.

## Ethics Statement

The studies involving human participants were reviewed and approved by Ethics Committee University Duisburg-Essen, Medical Faculty, Robert-Koch-Str. 9-11, 4514 Essen. Written informed consent for participation was not required for this study in accordance with the national legislation and the institutional requirements. Written informed consent was not obtained from the individual(s) for the publication of any potentially identifiable images or data included in this article.

## Author Contributions

TB organized and designed the table, wrote the manuscript, and was involved in primary patient´s care. VT, PM, FW, and HD were involved in primary patient´s care, data analysis and manuscript correction. ST and KS provided reagents, performed, and analyzed histology, discussed the data, and corrected the manuscript. DF initiated the series, was involved in patient care, designed, and corrected the manuscript. All authors contributed to the article and approved the submitted version.

## Conflict of Interest

The authors declare that the research was conducted in the absence of any commercial or financial relationships that could be construed as a potential conflict of interest.

## Publisher’s Note

All claims expressed in this article are solely those of the authors and do not necessarily represent those of their affiliated organizations, or those of the publisher, the editors and the reviewers. Any product that may be evaluated in this article, or claim that may be made by its manufacturer, is not guaranteed or endorsed by the publisher.
